# Inhibition of Protein Aggregation by Several Antioxidants

**DOI:** 10.1155/2018/8613209

**Published:** 2018-03-25

**Authors:** Samra Hasanbašić, Alma Jahić, Selma Berbić, Magda Tušek Žnidarič, Eva Žerovnik

**Affiliations:** ^1^Faculty of Pharmacy, Department of Biochemistry, University of Tuzla, Univerzitetska 1, 75000 Tuzla, Bosnia and Herzegovina; ^2^Jožef Stefan International Postgraduate School, Jamova 39, SI-1000 Ljubljana, Slovenia; ^3^Department of Biotechnology and Systems Biology, National Institute of Biology, Večna pot 111, SI-1000 Ljubljana, Slovenia; ^4^Department of Biochemistry and Molecular and Structural Biology, Jožef Stefan Institute, Jamova 39, SI-1000 Ljubljana, Slovenia; ^5^Center of Excellence for Integrated Approaches in Chemistry and Biology of Proteins (CipKeBip), Jamova 39, SI-1000 Ljubljana, Slovenia

## Abstract

Amyloid fibril formation is a shared property of all proteins; therefore, model proteins can be used to study this process. We measured protein aggregation of the model amyloid-forming protein stefin B in the presence and absence of several antioxidants. Amyloid fibril formation by stefin B was routinely induced at pH 5 and 10% TFE, at room temperature. The effects of antioxidants NAC, vitamin C, vitamin E, and the three polyphenols resveratrol, quercetin, and curcumin on the kinetics of fibril formation were followed using ThT fluorescence. Concomitantly, the morphology and amount of the aggregates and fibrils were checked by transmission electron microscopy (TEM). The concentration of the antioxidants was varied, and it was observed that different modes of action apply at low or high concentrations relative to the binding constant. In order to obtain more insight into the possible mode of binding, docking of NAC, vitamin C, and all three polyphenols was done to the monomeric form of stefin B.

## 1. Introduction

Properties of the amyloid state are generic to all proteins [1–3], whereas subtle differences in sequence and 3D structure dictate the propensity to form such ordered aggregates [4, 5]. Even though practically any protein can be induced to form the amyloid state *in vitro*, only about 50 of amyloidogenic proteins aggregate *in vivo* and cause disease [6, 7]. The aggregation of proteins finally leads to amyloid fibrils, which get sequestered into different inclusion bodies, whereas most dangerous proved soluble oligomers, which likely bind and perforate membranes causing toxicity. Therapy for neurodegenerative diseases, which would attack the roots of this pathological process and even cure the disease, is urgently needed, due to the aging population. Ways of how to stop the aberrant process of protein aggregation at a particular point are several [8]. One way is to support the native state by stabilizing antibodies, yet another is to recruit chaperone proteins or augment degradation pathways [9–14]. However, these new ways of possible early treatments are still under investigation in animal models and clinical trials.

Oxidative stress (OS) is one of the most important characteristics of neurodegenerative diseases [15]. It is known that it modifies proteins and causes their misfolding and aggregation. On the other side, protein aggregates bind divalent metal ions (Fe^2+^ and Cu^2+^), which in combination with hydrogen peroxide leads to the formation of reactive oxygen species (ROS) [16]. Therefore, neuroprotection by reducing protein aggregates or ROS or both seem achievable pharmacological targets [17]. However, very few effective compounds have been developed for clinical application and even fewer have been successful because of their toxicity and potential carcinogenicity. Natural antioxidants provide neuroprotective effects through a variety of biological actions, such as scavenging free radicals, interaction with transition metals, modulation of different enzymes and effects on intracellular signaling pathways, and gene expression [17]. Several epidemiological studies suggest that diets rich in antioxidants offer protection against numerous pathologies such as cancer, heart disease, hypertension, neurodegenerative diseases, and stroke [17, 18]. One should keep in mind that therapeutic use of relatively safe natural polyphenols is limited by their pharmacokinetics. In addition, these compounds can hardly pass the blood-brain barrier and reach an active concentration in the brain [17]. Some derivatives may act better; for example, it was shown that metal complexes of curcumin inhibited more potently the fibrillation of amyloid-beta (A*β*) than the parent compound did [19].

**Figure 1:**
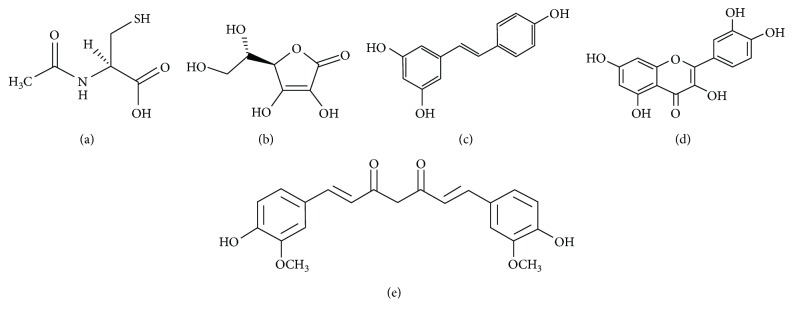
Structures of (a) NAC, (b) vitamin C, (c) resveratrol, (d) quercetin, and (e) curcumin. Computed chemical and physical properties are given in Supplementary Table S2.

Knowing that protein aggregation is a shared property of all proteins, model proteins can be used to study this process. We have studied many facets of oligomers and amyloid-like fibril formation by human stefin B [20–25]. Here, we use this system to study the effect of various antioxidant substances on the kinetics, yield, and morphology of amyloid fibril formation. For this study, we have chosen vitamins C (vit C) and E (vit E), *N*-acetyl cysteine (NAC), and three polyphenols: curcumin (Cur), resveratrol (Res), and quercetin (Quer) (Figure 1). Vitamins C and E and NAC, as well as polyphenolic compounds, reduce reactive oxygen species by their free electron scavenging action [26]. However, they also may directly interact with a protein hydrogen-bonding network or aromatic residues, respectively, with an impact on protein aggregation [27, 28].

NAC ((2R)-2-acetamido-3-sulfanyl propanoic acid) (Figure 1(a)) has been shown to be an effective precursor to glutathione (GSH) production, and it is known to cross the blood-brain barrier (BBB) [29]. It provides cysteine, which is the rate-limiting substrate in glutathione synthesis. Therefore, it acts as an antioxidant by increasing GSH levels and by directly interacting with free radicals [29]. Its ability to effectively disrupt the fibrillogenesis of the A*β* peptide has already been reported [30]. Vitamin C ((2R)-2-[(1S)-1,2-dihydroxyethyl]-3,4-dihydroxy-2H-furan-5-one) is believed to be a vital antioxidant in the brain (Figure 1(b)) [31]. Namely, a huge body of evidence suggests that vitamin C may change the course of neurological diseases and serve as a potential therapeutic tool. Intracellularly, it helps to maintain several key processes, including neuronal maturation and differentiation, myelin formation, synthesis of catecholamine, modulation of neurotransmission, and antioxidant protection [31]. Targeted deletion of the sodium-vitamin C cotransporter in mice resulted in widespread cerebral hemorrhage and death [31].

Polyphenols are secondary plant metabolites characterized by aromatic rings and one or more hydroxyl groups with different structural complexities (Figures 1(c)–1(e)). The most abundant class of phenolic compounds in plants includes flavonoids, such as flavonols, flavones, isoflavones, and anthocyanidins. Resveratrol (3,5,40-trihydroxystilbene) (Figure 1(c)) is an abundant polyphenol, a phytoalexin present in red wine and grapes. It has two phenolic rings connected by a double bond and has two isoforms *trans*-resveratrol and *cis*-resveratrol. *trans*-Resveratrol is believed to be responsible for the French paradox [32, 33]. A huge body of evidence shows its effects on the amyloid fibrillation process [34–37]. One of the most common dietary polyphenols is flavonol quercetin (2-(3,4-dihydroxyphenyl)-3,5,7-trihydroxy-4H-chromen-4-one) (Figure 1(d)). Quercetin is widely present in apples, tea, capers, and onions. Its inhibitory activity toward amyloid fibrillation has already been reported [38–41]. Curcumin ((1E,6E)-1,7-bis(4-hydroxy-3-methoxyphenyl)hepta-1,6-diene-3,5-dione) is a yellow pigment present in spice turmeric (lat. *Curcuma longa*) (Figure 1(e)). According to over 6000 citations, it has been associated with many beneficient activities, such as antiamyloidogenic, antioxidant, anti-inflammatory, anticancer, antiviral, and antibacterial [42, 43]. On top of that, over one hundred clinical studies have been carried out with curcumin. Searching the literature, curcumin has been shown to inhibit amyloid fibrillation of fA*β* [44, 45], prion protein [46], insulin [47], hen egg white [48], lysozyme [49, 50], and *β*-lactoglobulin [51]. Unfortunately, its wide application is limited due to its pharmacokinetics, which disfavors its bioavailability. Various formulations of curcumin that are currently available and ongoing studies should help to overcome this problem [42].

## 2. Materials and Methods

### 2.1. Materials

In this study, we have used human stefin B (stB wt) as a model protein. This recombinant protein has Cys 3 replaced with Ser. 2,2,2-Trifluoroethanol (TFE) was purchased from Fluka, Thioflavin T (ThT) from Aldrich, and bis(sulfosuccinimidyl)suberate (BS^3^) from Thermo Fisher Scientific. Other chemicals were from Sigma, Carlo Erba, Serva, and Merck.

### 2.2. Expression and Purification

Expression and purification have already been described elsewhere [52]. Briefly, DNA constructs were transformed into the BL21(DE3)pLysS strain of *E. coli*. Expression was induced with IPTG (final concentration 1 mM). Three hours after induction, cells were separated from the medium and lysed. Expression efficiency was checked by SDS-PAGE electrophoresis. Cell lysates were additionally purified by adding 4% polyethyleneimine (PEI) and repeated centrifugation. This way, most of the contaminants such as nucleic acids and most bacterial (predominantly acidic) proteins were removed from the lysate. The stB wt was isolated from purified cell lysates by affinity chromatography on carboxymethyl (CM)-papain-Sepharose. The nonspecifically bound material was eluted with 0.01 M Tris-HCl containing 0.5 M NaCl at pH 8.0. The wt stefin B was eluted with 0.02 M TEA buffer at pH 10.5. Ionic strength and pH were immediately adjusted with strong 0.2 M phosphate buffer, pH 7, with 1 M NaCl leading to fast refolding. Additional purification was done using SEC on Sephacryl S-200 (Amersham Pharmacia Biotech) equilibrated with 0.01 M phosphate buffer, containing 0.12 M NaCl at pH 6.1. Purity was checked by SDS-PAGE electrophoresis.

### 2.3. ThT Fluorescence

ThT dye was used to determine the presence of amyloid fibrils. Fluorescence was measured using a PerkinElmer model LS-50B luminescence spectrometer. Excitation was set to 440 nm, and spectra were recorded from 455 nm to 600 nm. ThT dye was dissolved in phosphate buffer (25 mM, 0.1 M NaCl at pH 7.5) at 15 *μ*M (*A*_416_ = 0.66). Fibrils were grown under mild conditions at pH ~5 (0.015 M acetate buffer, 0.15 M NaCl at pH 4.8) at room temperature; protein concentrations were 34 *μ*M. In order to accelerate fibril formation, fibrillation mixtures contained 10% v/v TFE. Fifty microliters of the protein solution in which fibrils were growing was added to 570 *μ*L of the ThT buffer just before measurement. A fresh ThT probe was prepared daily. ThT fluorescence was measured using a 0.5 cm cuvette at 25°C. Excitation and emission slits were set at 5 nm and 7 nm, respectively. Data were collected every 0.5 nm. Fluorescence intensities at 482 nm were plotted against time. Each measurement was performed at least twice in duplicates, and the mean value is presented (average of duplicate measurements ± standard deviations). The blind probe was followed for each antioxidant and subtracted to get final values.

### 2.4. Transmission Electron Microscopy

Protein samples (15 *μ*L of 34 *μ*M protein solution) were applied on a Formvar- and carbon-coated grid. After 3 min, the sample was soaked away and stained with 1% (w/v) uranyl acetate. Samples were observed with a Philips CM100 (FEI, Netherlands) transmission electron microscope operating at 80 kV. Images were recorded using a Bioscan or ORIUS SC 200 CCD camera (Gatan Inc., Washington, DC, USA), using the DigitalMicrograph software (Gatan Inc., Washington, DC, USA). Two parallel grids were prepared for each sample, at least 10 grid squares were inspected thoroughly, and many micrographs were taken of each grid.

### 2.5. Molecular Docking

The molecular docking study was performed using the SwissDock server http://www.swissdock.ch/. SwissDock is based on the docking software EADock DSS, whose algorithm consists of many steps [53]. The target molecules were provided as PDB files (stB wt PDB id: 4N6V). Chain A of the protein was selected, water molecules and ions were removed, and all hydrogen atoms were added. A uniform procedure led to numerous predictions for each chosen ligand. Binding modes are scored using FullFitness and clustered. Clusters are then ranked using FullFitness of their elements. In consecutive cycles, the structure of the lowest “FullFitness” and estimated ΔG value was selected and the neighboring docked ligand structures were collected as representative. In other words, only the minimum energy conformation states of the ligand-bound protein complex out of many generated binding modes were considered. Binding modes were visualized in JSmol, and PDB was used for the identification of residues involved in binding.

### 2.6. ThT Fluorescence Bias Measurement

Fluorescence measurements were done using a TECAN Safire plate reader (Thermo Fisher Scientific) in 96 wells at 25°C. Solutions of stB wt preformed fibrils were incubated at room temperature in the absence and presence of different final concentrations of antioxidants. An 11.4-fold volume excess of ThT was added to each well prior to fluorescence reading. For each assay, the fluorescence of antioxidant without stB wt with ThT dye was also monitored. The excitation wavelength was set at 440 nm, and the emission wavelength was set from 455 to 600 nm. The emission wavelength step size was 1 nm, and excitation and emission bandwidths were set at 7.5 nm. The fluorescence intensity at 482 nm was read. Each sample was followed in triplicate, and the mean value is presented.

### 2.7. Circular Dichroism

Far-UV circular dichroism (CD) spectra were measured at room temperature by using a Circular Dichroism Spectrometer MOS-500 (Bio-Logic Science Instruments). The fibrillation mixture was prepared as described in Section 2.3. Protein concentration was 34 *μ*M (*A*_280_ = 0.15). The temperature was maintained at 25°C throughout. A 1 mm quartz cuvette was used for all CD spectra. Data were recorded from 250 to 200 nm with a 1 nm sampling interval. The final spectra were the average of three repeated experiments, and the background (the CD spectrum of the sample without stB wt and antioxidants) was subtracted.

### 2.8. SDS-PAGE Electrophoresis and Cross-Linking

A fibrillation mixture of stB wt was prepared as described in Section 2.3. Samples aged 24 hours were applied to SDS-PAGE gel using the standard procedure. To make sure that different higher molecular species will remain stable in the presence of SDS and high temperature, we performed cross-linking with BS^3^ as a cross-linker. Instructions from the manufacturer were followed, and 50-fold molar excess of the cross-linker has been used. stB wt fibrillation mixture in the absence and presence of the cross-linker was used as control.

### 2.9. Steady-State Fluorescence Quenching Measurements

Steady-state fluorescence quenching measurements of stB wt in the presence of different concentrations of quenchers, that is, antioxidants, were done using the TECAN Safire plate reader (Thermo Fisher Scientific) in 96 wells at 25°C. The protein concentration was 34 *μ*M, and antioxidant concentrations were varied. Intrinsic tyrosine fluorescence was measured by exciting protein at 277 nm, and emission spectra were recorded in the range of 290 to 360 nm. The excitation and emission slit widths were set at 7.5 nm, whereas emission wavelength step size was set to 1 nm. Each measurement was done in triplicate. The data were analyzed according to the Stern-Volmer equation:

(1) $$\begin{array}{c} \frac{F_{o}}{F}=K_{sv}\left[Q\right]+1, \end{array}$$

where *F*_o_ and *F* are the fluorescence intensities in the absence and presence of quenchers, that is, antioxidants, and *K*_sv_ is the Stern-Volmer quenching constant. Binding constants and binding sites were obtained from (2), which is basically a modified Stern-Volmer equation:

(2) $$\begin{array}{c} \log\left(F_{o}-\frac{F}{F}\right)=\log K_{a}+n\log\left[Q\right], \end{array}$$

where *K*_a_ is the association constant and *n* is the number of binding sites [54].

## 3. Results

The main goal of our study was to assess the effects of different antioxidants on the fibrillation profile of our model protein—human stefin B (stB). Therefore, we have chosen three polyphenols and two compounds with a more simple structure (Figure 1). Firstly, their effects on the amyloid fibrillation profile of stB were followed using ThT fluorescence measurements. It is a usual tool as ThT fluorescence increases in the presence of amyloid fibrils and hence is usually used to characterize inhibitors of amyloid fibrillation reaction. However, precaution is obligatory because ThT fluorescence can be quenched by polyphenol compounds [55–57]. As a complementary tool, TEM images were recorded in the *plateau* phase of the reaction. As described in the Methods section, for each sample, 2 grids were prepared and many images of each were observed to obtain an estimate of the yield of amyloid fibrils. TEM data mostly support ThT fluorescence results; however, when quenching was indicated, we measured the ThT fluorescence bias (Supplementary Figure S1). Furthermore, molecular docking was performed in order to obtain predictions of the binding mode for each of the antioxidants. Additional data regarding binding constants were provided using steady-state fluorescence quenching measurements and Stern-Volmer constants. Alterations of the secondary structure were checked using circular dichroism. The oligomeric state was determined using cross-linking prior to SDS-PAGE electrophoresis. Results are described in this section and analyzed in the Discussion section. One part of the results is presented and elaborated in Supplementary materials.

**Figure 2:**
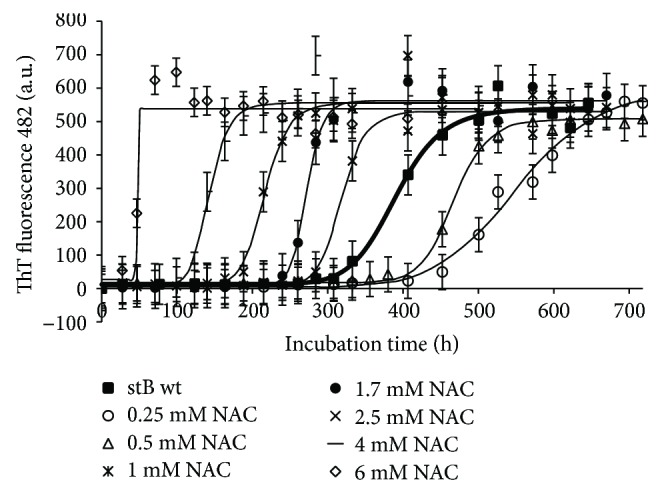
Aggregation kinetics of stefin B monitored by ThT binding/fluorescence in the absence and presence of different concentrations of NAC. ThT fluorescence emission at 482 nm was monitored upon excitation at 440 nm. Protein concentration was 34 *μ*M, and concentrations of NAC were varied. Each sample was incubated at room temperature in 0.015 M acetate buffer, 0.15 M NaCl at pH 4.8, prior to mixing with the ThT probe as described in Section 2.

**Figure 3:**
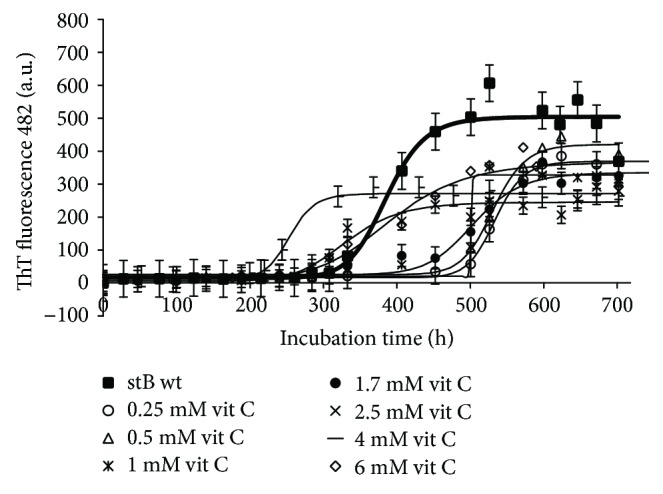
Aggregation kinetics of stefin B monitored by ThT binding/fluorescence in the absence and presence of different concentrations of vitamin C (vit C). ThT fluorescence emission at 482 nm was monitored upon excitation at 440 nm. Protein concentration was 34 *μ*M, and a concentration of vit C was varied. Each sample was incubated at room temperature in 0.015 M acetate buffer, 0.15 M NaCl at pH 4.8, prior to mixing with the ThT probe as described in Section 2.

**Figure 4:**
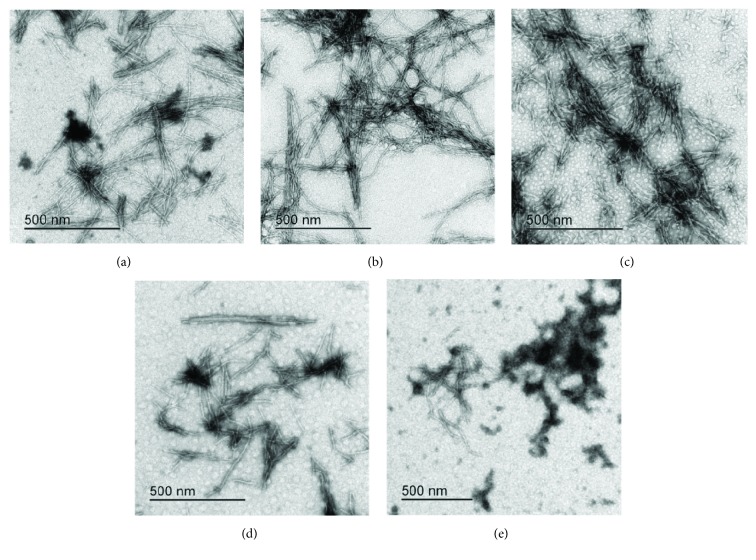
TEM data at the *plateau* of the fibrillation reaction by stefin B. Control: (a) stB in water. Samples: (b) stB with NAC at 4.0 mM and (c) stB with vitamin C at 4.0 mM, (d) 2.5 mM, and (e) 0.25 mM.

Results of the concentration dependence of NAC and vitamin C on fibril growth as judged by ThT fluorescence are gathered in Figures 2 and 3. In Figure 2, it can be seen that NAC from 1 to 6 mM concentrations (average molar ratio 100 : 1 NAC to protein) promotes protein aggregation, that is, it reduces the lag phase. The final ThT fluorescence intensity does not change substantially (Figure 2); neither does TEM show influence on the final yield or morphology of the mature amyloid fibrils (Figure 4(b) shows 4.0 mM NAC). Lower concentrations, that is, 0.25 mM and 0.5 mM NAC, inhibit fibril growth, that is, prolong the lag phase.

In Figure 3, it can be seen that vitamin C at 2.5 mM and 4 mM concentrations also accelerates the reaction of amyloid fibril formation, that is, shortens the lag phase and lowers the final amount of mature fibrils as judged by ThT fluorescence intensity (Figure 3). This lowering of fluorescence may be partially due to a quenching effect as TEM does not show a lower quantity of mature fibrils at 4 mM or 2.5 mM vitamin C concentrations (Figures 4(c) and 4(d)). At lower concentration, vitamin C acts similarly to NAC; the lag phase is prolonged. Noteworthily, vitamin C at the 0.25 mM concentration inhibits fibril growth, that is, prolongs the lag phase (Figure 3) and lowers the mass of fibrils on account of the aggregates as shown by TEM (Figure 4(e)).

We have also measured the effect of vitamin E dissolved in ethanol. No significant effect of this vitamin either on the lag phase or on ThT intensity was observed (data not shown). Fibrils were not inhibited in this case (TEM data not shown).

**Figure 5:**
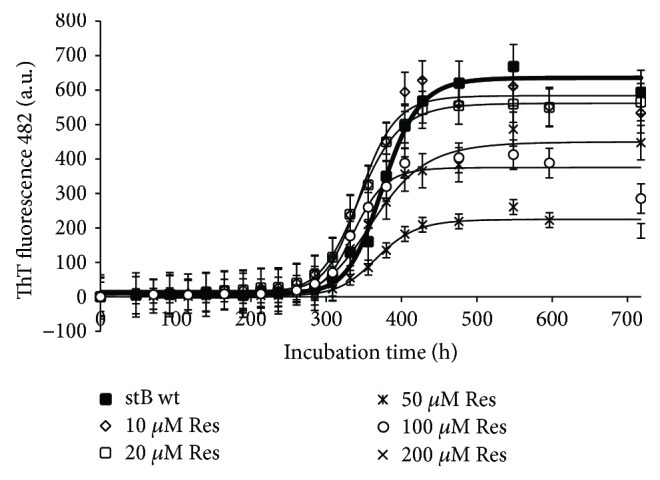
Effect of resveratrol on amyloid fibril formation by stefin B. Aggregation kinetics monitored by ThT binding in the absence and presence of different concentrations of Res. The inhibitory effect on amyloid formation of stB wt was monitored by following the ThT fluorescence emission at 482 nm upon excitation at 440 nm. Concomitantly, the aggregation behavior of stB wt with final 1% v/v DMSO was followed each day as a control because Res was dissolved in DMSO to a final 1% v/v. Protein concentration was 34 *μ*M, and concentrations of Res were varied.

**Figure 6 fig6:**
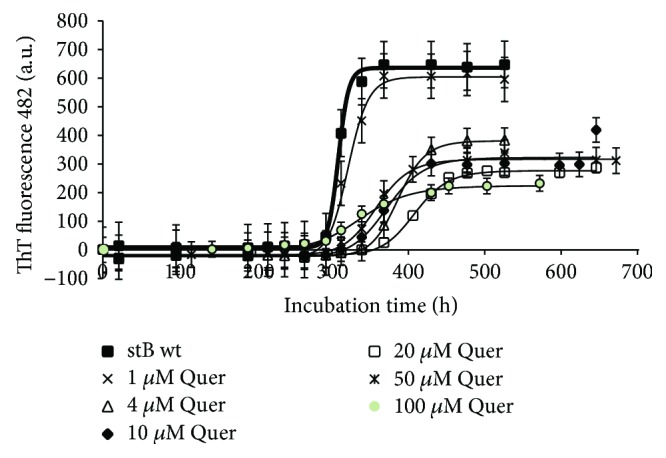
Effect of quercetin on amyloid fibril formation by stefin B. Measurement was done by following ThT fluorescence—as described in Figure 5.

**Figure 7:**
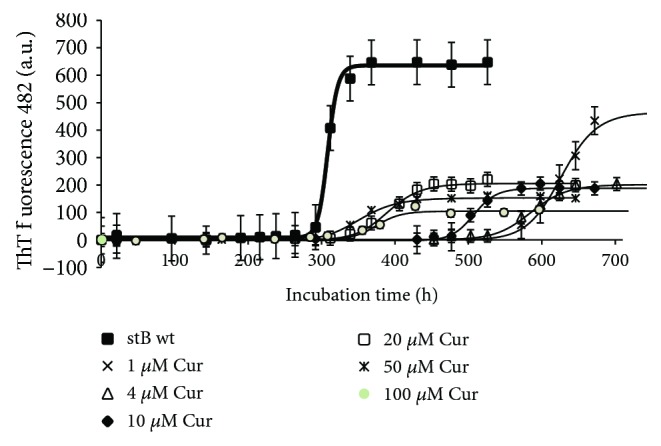
Effect of curcumin on amyloid fibril formation by stefin B. Measurement was done by following ThT fluorescence—as described in Figure 5.

**Figure 8:**
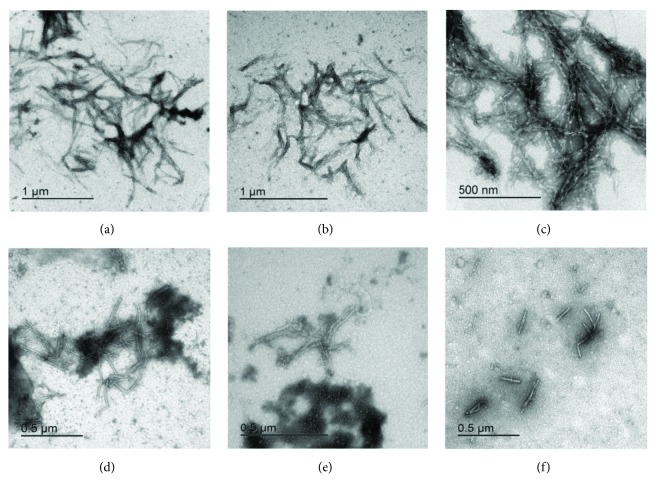
TEM data collected at the *plateau* of the reactions of amyloid fibril formation by stefin B in the presence and absence of polyphenols. Controls (a), (b), and (c) show stB in (a) water, (b) in 1% DMSO, and (c) in 1% ethanol in comparison to 50 *μ*M concentrations of (d) resveratrol, (e) quercetin, and (f) curcumin.

**Figure 9:**
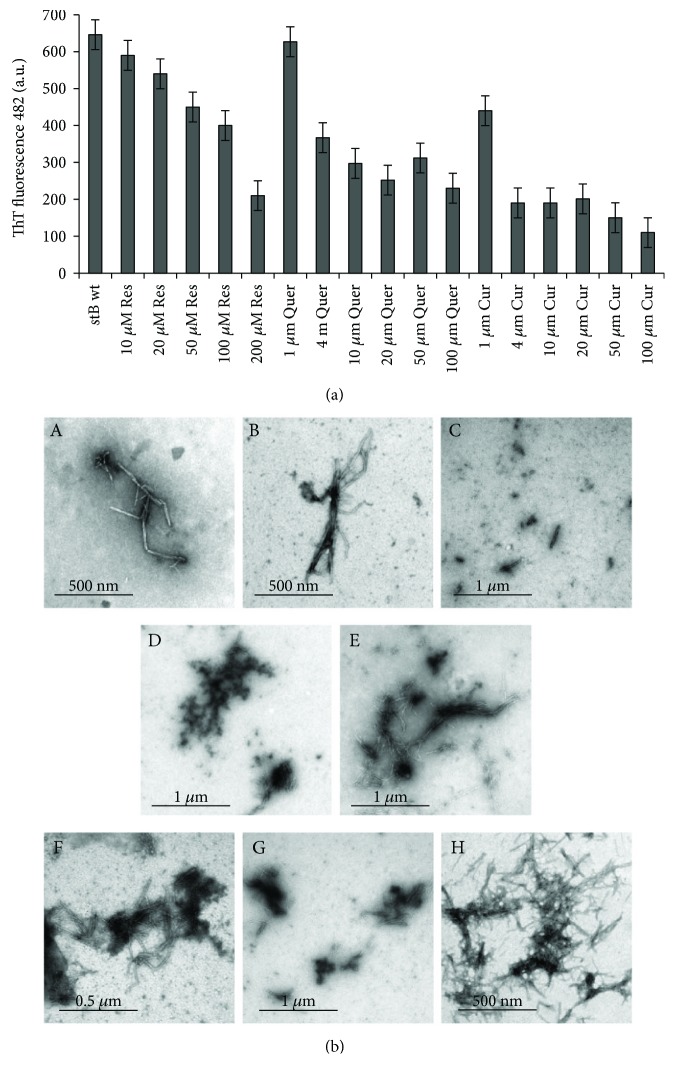
ThT fluorescence intensity versus TEM data. (a) Graph of ThT fluorescence intensity in the *plateau* phase of amyloid fibrillation versus antioxidant concentration. (b) TEM data. Concentration dependence of fibril morphology and approximate amounts; samples were taken in the *plateau* phase of the reactions of amyloid fibril formation by stB at different polyphenol concentrations: (A) 1 *μ*M Cur, (B) 10 *μ*M Cur, (C) 50 *μ*M Cur, (D) 1 *μ*M Quer, (E) 50 *μ*M Quer, (F) 50 *μ*M Res, (G) 100 *μ*M Res, and (H) 200 *μ*M Res.

Results of different concentrations of the three polyphenols resveratrol, quercetin, and curcumin on ThT fluorescence are shown in Figures 5 [Fig fig6]–7. Resveratrol (Figure 5) shows no significant effect on the lag phase, whereas ThT fluorescence intensity slightly decreases in a concentration-dependent manner. However, TEM data (Figures 8(d) and 9(b), F–H) show more aggregates remaining, pointing to a quenching effect (Supplementary Figure S1). Quercetin (Figure 6) prolongs the lag phase; however, this effect is less expressed than for curcumin (Figure 7). Interestingly, when compared to other antioxidants, quercetin shows a different behavior. According to TEM results, more aggregates remain and, in some regions, fibrils look more amorphous (Figure 8(e)). Curcumin affects the lag phase the most (Figure 7), and it also reduces the final ThT fluorescence the most. However, this observation may be due to a strong quenching effect (Supplementary Figure S1). In order to assess the differences in the final quantity of fibrils at certain curcumin concentrations, we have to rely on TEM (Figures 8(f) and 9(b), A–C). This method is not meant to test the amount of the fibrils quantitatively; many grids have to be observed until statistically valid amounts can be estimated. Still, it enables us to estimate the effects of different antioxidants on the final yield and morphology of the aggregates, and the relation between the forms.

We have also measured how the concentration dependence of the three polyphenolic antioxidants, that is, Res, Quer, and Cur, reflects on ThT fluorescence measured at the lag phase of stB fibrillation (Figure 9(a)) as compared to TEM (Figure 9(b)). It can be seen that ThT fluorescence intensity is inversely proportional to the concentration of Res, that is, fluorescence decreases as the concentration of the antioxidant increases (Figure 9(a)). Quer and Cur act similarly—the lowest concentration of both antioxidants did not affect amyloid fibrillation at all or the effect was minor in the case of 1 *μ*M Cur, whereas higher concentrations caused a reduction in fluorescence intensity. However, this effect was more pronounced in the case of Cur (Figure 9(a)). TEM images partially reflect these results (Figure 9(b)). According to TEM results, the final quantity of fibrils in the presence of higher concentrations of Cur (Figure 9(b), C) is lower than in the presence of lower concentrations (Figure 9(b), A). Effects of Quer are specific (Figure 9(b), D and E), and many aggregates are visible in the case of both concentrations. When compared to control, fibrils are shorter and thicker and appear sticky when Cur or Quer is present. TEM data for Res partly support ThT fluorescence results (Figure 9(b), F–H); the inhibitory effect of 100 *μ*M Res is more obvious than that of 50 *μ*M Res. In the case of 200 *μ*M Res, more fibrils are visible.

CD spectra in the far-UV region have not shown any significant change in the secondary structure of stB wt samples aged 24 hours at given concentrations of antioxidants (Supplementary Figure S2). In order to determine the oligomeric state, the fibrillation mixture was subjected to FPLC analysis on Superdex 75 and cross-linking prior to application on the SDS-PAGE gel. The FPLC elution profile could not be used as a reliable result because the protein severely aggregated in the presence of different antioxidants (data not shown). However, SDS-PAGE electrophoresis after cross-linking with BS^3^ has shown that antioxidants shift the equilibrium to higher forms, such as tetramers and higher oligomers (Supplementary Figure S3. Tetramers and higher oligomers are marked with an arrow). According to the gel, when protein is exposed to antioxidants, there are more dimers as well, than in the case of the control (Supplementary Figure S3).

**Figure 10:**
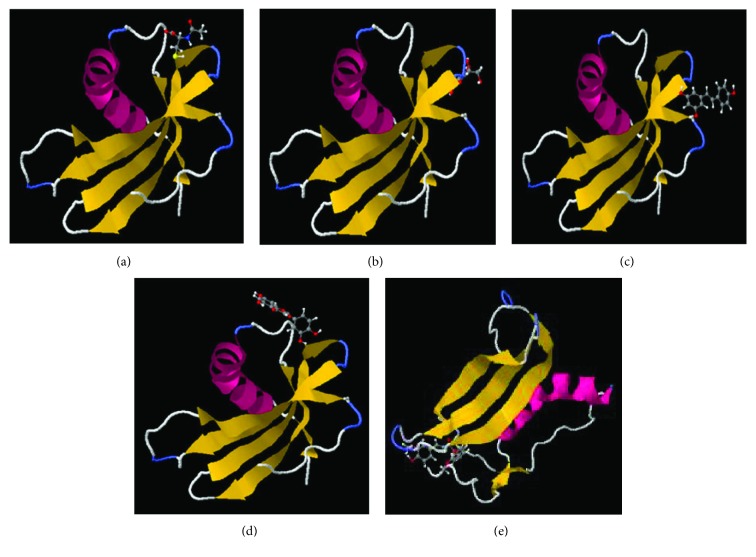
Binding predictions obtained by molecular docking. Molecular docking was performed using SwissDock, and the structures of the most stable stB complexes with (a) NAC, (b) vitamin C, (c) resveratrol, (d) quercetin, and (e) curcumin were selected.

A molecular docking study has offered predictions of binding modes for each antioxidant (Figure 10). Thanks to SwissDock, it was possible to obtain docking prediction in JSmol and binding parameters based on the inserted PDB code for stB and the specific structure of the antioxidant. Databank was used to determine amino acid residues that participate in specific protein-ligand interactions (Supplementary Table S1). Interestingly, the program has offered many prediction variants for each antioxidant with the exception of curcumin; this antioxidant has each time been positioned within the same pocket (Figure 10(e), Supplementary Table S1). This observation is supported by results of intrinsic Tyr fluorescence measurements (Supplementary Figures S4E and S5B, D). Supplementary Figure S4E shows a significant decrease in Tyr fluorescence intensity in a concentration-dependent manner, and Stern-Volmer plots and binding constants show that curcumin binds more tightly than do other antioxidants studied here (Supplementary Figure S5B, D).

## 4. Discussion

Protein self-assembly into amyloid fibrillary state is characteristic of numerous debilitating diseases [2]. It is known that natural organic dyes, for example, Congo Red, bind tightly to proteins and hence block their self-assembly. The ability of this and other molecules to prevent amyloid accumulation has generated an immense interest in elucidating their key structural properties which contribute to inhibitory potency [34, 58, 59]. It is clear that a better understanding of the structure-activity relationships would facilitate the creation of new protein aggregation inhibitors. In turn, these insights might be crucial for deciphering the key elements of the amyloid fibrillation puzzle. Thus, the main goal of this study was to understand at which point the chosen compounds influence protein aggregation reaction and which structural features of the compounds may explain their antiamyloid activity toward our model protein—human stefin B.

The idea that some polyphenolic compounds may interfere with protein aggregation is not new. So far, it is well known that ligands which interfere with amyloid fibrils are flat, planar molecules with substituted aromatic end groups [60]. The first such mention dates back to 2004 when the authors Ono et al. examined the effects of curcumin and rosmarinic acid (RA) on the formation, extension, and destabilization of fA*β*(1–40) and fA*β*(1–42) [61]. Cur and RA dose-dependently inhibited fA*β* formation from A*β*(1–40) and A*β*(1–42), as well as their extension. In addition, they dose-dependently destabilized preformed fA*β*s [61]. Moreover, in 2006 Porat et al. suggested an additional mechanism of Cur action [28]. By then, the inhibition mechanism of polyphenol antioxidants had been mostly considered as a result of their antioxidative properties. Taking into consideration that polyphenols are capable of inhibiting amyloid fibril formation *in vitro* and in view of their structural similarities, these authors proposed an additional mechanism of action. They suggested that both structural constraints and specific aromatic interactions are important for the inhibition of amyloid fibril formation as they provide proper positioning of the polyphenol inhibitors in the amyloidogenic core.

Our study aimed at showing the effect of various antioxidant substances on the kinetics, yield, and morphology of amyloid fibrils formed by human stefin B *in vitro*. We believe that it mimics other similar systems. To follow the amount of amyloid fibrils, ThT fluorescence measurement was used. It is a usual tool as ThT fluorescence increases in the presence of amyloid fibrils and hence can be used to characterize inhibitors of amyloid fibrillation reaction. However, this assay can be biased by the presence of exogenous compounds, in our case polyphenols, NAC, and vitamin C. There is a study on the interference of the three polyphenols to the intensity of ThT fluorescence [55]. In that study, authors have shown that when it comes to quantification of amyloid fibril formation in the presence of polyphenols, ThT fluorescence should be interpreted with caution. In other words, such compounds can significantly bias ThT fluorescence due to a quenching effect [55]. Therefore, we have checked the quenching properties of each of the chosen antioxidant. Supplementary Figure S1 shows that each compound acts as a quencher of ThT fluorescence. However, results obtained by TEM and the differences in the lag time in ThT fluorescence are still valid to evaluate the inhibitory effects of these compounds on protein aggregation to amyloid fibrils and therefore worth discussing.

ThT fluorescence measurements have not detected any major decrease in the intensity when different concentrations of NAC were added to the stB fibrillation mixture. TEM images similarly did not show any significant difference in the final yield and morphology of stB fibrils (Figure 2 and 4(b)). On the other hand, the lag phase varied in a concentration-dependent manner, that is, higher concentrations have shortened the lag phase, whereas lower concentrations have prolonged it (Figure 2). Noteworthily, a similar behavioral pattern can be observed in the case of vit C (Figure 3), which can probably be explained as their significant influence on the microenvironment of the amyloidogenic core. This observation is not due to pH change because, in our hands, antioxidants did not alter pH. Moreover, electrophoretic techniques can help in determining oligomer sizes in the fibrillation mixture [62]. In our study, SDS-PAGE electrophoresis upon cross-linking has shown that antioxidants NAC and vit C shift the equilibrium to higher forms such as tetramers and higher oligomers (Figure S3; tetramers with a molecular mass of ~44 kDa and higher oligomers are marked with arrows). Similar observations have already been described by other authors [63, 64]. According to the gel, there are more dimers (molecular mass ~22 kDa, marked with an arrow) than in the case of the control (Figure S3). Higher oligomers appear only when protein is exposed to very high concentrations of antioxidants, in our case 2.5 mM and 6 mM vit C (Figure S3, lanes 6 and 7) and 2.5 mM and 6 mM NAC (Figure S3, lanes 9 and 10). Of note, the highest concentrations of both NAC and vit C decreased the lag phase length (Figures 2 and 3).

If we compare our data to the effect of vitamin C to amyloid fibril formation of *β*-lactoglobulin [27], a similar inhibitory effect is observed. Vitamin C inhibits the fibril formation of stefin B in the range of concentrations from 0.25 mM to 0.5 mM, both by prolonging the lag phase (Figure 3) and by diminishing the final amount of the fibrils, as judged by TEM (Figure 4(e)). Note that ThT may be prone to quenching effects; therefore, one should dissect whether lower fluorescence indeed means a lower amount of the fibrils [55]. Vitamin C at higher concentrations than 0.5 mM (Figure 3) decreases the lag phase and promotes aggregation, similarly to NAC. Final ThT fluorescence appears much lower whereas TEM shows a similar amount of the fibrils (Figures 4(c) and 4(d)). Here, the discrepancy can best be explained by a severe quenching effect on ThT fluorescence. Lee et al. explained the inhibitory effect of vitamin C through its interference with exposed hydrogen atoms of the N-H groups in the *β*-sheet backbone [27]. To be specific, metabolites of vitamin C, which are generated in the aqueous solution, such as ascorbate anions and dehydroascorbic acid, can shield electrostatic interactions between *β*-sheets due to their specific interactions and cause disruption of *β*-sheet stacking. This mechanism is different from antioxidation, which is usually considered as a major mechanism of vitamin C action. It was even reported that it can decrease amyloid plaque burden in the cortex and hippocampus when tested on cross-bred mice [65]. In our study, it was confirmed once more that vit C has potential as an antiamyloid substance. However, at higher concentrations it may be less beneficial as it increases the most toxic higher oligomers.

The three polyphenols each behave differently. Curcumin at all concentrations inhibited the amyloid fibril formation (Figure 7). Lower concentrations have a greater effect on the prolongation of the lag phase; however, the final mass of the fibrils appears higher. At the concentration of 50 *μ*M, curcumin both prolongs the lag phase and reduces the amount of mature fibrils. The quantity and morphology of the fibrils at 50 *μ*M Cur were checked by TEM and are presented in Figure 8(f). With curcumin, one cannot neglect a quenching effect; therefore, TEM results are more relevant than that using ThT fluorescence. TEM data in Figure 8(f) confirm that curcumin at 50 *μ*M inhibits fibril growth. Noteworthily, the effect follows a concentration-dependent pattern: at lower concentrations, such as 1 *μ*M Cur, the fibrils grow longer, whereas at 50 *μ*M Cur the fibrils are shorter and fewer (Figure 9(b), A–C). Resveratrol does not reduce the lag phase (Figure 5). It apparently reduces the final yield of the fibrils (Figure 5), which however was not confirmed by TEM (Figure 8(d)). Quercetin slightly prolongs the lag phase (Figure 6). However, more aggregates are produced than the fibrils as can be seen by TEM (Figures 8(e) and 9(b), D and E).

Docking studies can provide us with important information and are a very useful tool for understanding the prevailing binding modes between protein and ligands. In order to investigate the possible mode of interaction and determine the most stable complex between stefin B and chosen antioxidants, docking using SwissDock was performed (Figure 10). Docking investigation has offered about 100 different variants for each ligand, and those with the lowest values for free binding energy were chosen (Supplementary Table S1). Interestingly, different positions with different ΔG values were suggested for each ligand except for curcumin, which was each time positioned in a similar way and had the lowest overall ΔG value, as well (Figure 10). When compared to other ligands included in this study, curcumin has a specific chemical scaffold (Figure 1(e)); it contains two substituted aromatic groups symmetrically bound by a short carbohydrate chain. More discussion about why curcumin might be the strongest binder is given in the Supplementary Materials.

As a supporting data for docking study, Supplementary Figure S4 shows that each compound also acts as a quencher of tyrosine fluorescence. The observed reduction of fluorescence intensity indicates that each ligand binds in the vicinity of the tyrosine residue—as they come closer to the Tyr residues in the process of binding, their fluorescence intensity is being reduced. Stefin B is a multityrosine protein as it has three tyrosine residues (Tyr53, Tyr85, and Tyr97). Therefore, at first, it might be a complicated task to clarify which tyrosine could be accessible for the ligands. However, this question can be resolved using docking predictions and the human stefin B structure obtained using X-ray diffraction. More information about amino acid residues which participate in binding is given in Supplementary Table S1.

To conclude, in this study, we have studied the effect of 5 different antioxidant compounds on amyloid fibrillation of stefin B. Our results are mostly in line with other similar studies, showing that antioxidants with flat aromatic structures, as well as vit C and NAC, can interact with the aggregating protein and inhibit amyloid fibril formation at different stages. The current study highlights and partly confirms the possibility that antioxidant compounds can also fight the formation of toxic oligomers and amyloid fibrils. However, their ROS scavenging effect cannot be neglected. Considering that protein misfolding and aggregation cause many debilitating diseases, future studies are warranted.

## Supplementary Materials

Supplementary Materials Figure S1: effect of Res, Quer, and Cur and vit C on in situ real-time ThT fluorescence. Figure S2: the CD spectra of stB wt assemblies aged 24 hours in the absence and presence of 50 *μ*M Res. Figure S3: SDS-PAGE of stB wt assemblies aged 24 hours in the absence and presence of different concentrations of antioxidants; samples apart from those in lane 1 were cross-linked before SDS-PAGE. Table S1: molecular docking parameters of the antioxidant-protein interaction. Table S2: computed chemical and physical properties from https://pubchem.ncbi.nlm.nih.gov/. Figure S4: steady-state fluorescence quenching measurements of stB wt in the presence of different concentrations of quenchers, that is, antioxidants. Figure S5: Stern-Volmer plots and modified Stern-Volmer plots of the fluorescence quenching constant for antioxidant-protein complexes.
