# Supplementary material for: Inhibition of Protein Aggregation by Several Antioxidants
Source: Oxid Med Cell Longev. 2018 Mar 25;2018:8613209. doi: 10.1155/2018/8613209 (PMC5889867; doi:10.1155/2018/8613209)
Supplement: Supplementary Materials — Figure S1: effect of Res, Quer, and Cur and vit C on in situ real-time ThT fluorescence. Figure S2: the CD spectra of stB wt assemblies aged 24 hours in the absence and presence of 50 μM Res. Figure S3: SDS-PAGE of stB wt assemblies aged 24 hours in the absence and presence of different concentrations of antioxidants; samples apart from those in lane 1 were cross-linked before SDS-PAGE. Table S1: molecular docking parameters of the antioxidant-protein interaction. Table S2: computed chemical and physical properties from https://pubchem.ncbi.nlm.nih.gov/. Figure S4: steady-state fluorescence quenching measurements of stB wt in the presence of different concentrations of quenchers, that is, antioxidants. Figure S5: Stern-Volmer plots and modified Stern-Volmer plots of the fluorescence quenching constant for antioxidant-protein complexes. [file 8613209.f1.docx]

**SUPPLEMENTARY DATA**

In order to test the reliability of ThT fluorescence as a marker for amyloid fibril formation, ThT quenching effect was checked for each antioxidant in different concentrations (Figure S1). According to these results, every antioxidant affects fluorescence intensity severely. However, it should be noted that the procedure was not exactly the same. Namely, as described in section 2, fibrillation samples were prepared from protein, TFE, and antioxidant using acetate buffer. ThT fluorescence was measured daily in such a way that the fresh ThT was added to a fibrillation mixture. Quenching effect was determined in a slightly different manner; the antioxidants and ThT dye were added to preformed fibrils of stB wt which could possibly change the effect of antioxidants on fibril formation. In this case, the quenching effect might be result of polyphenols competition with ThT on preformed fibrils. However, TEM was used for obtaining estimates of fibril amount regardless of the apparent ThT fluorescence intensity.

**Figure S1.** **Effect of the Res, Quer, Cur and vit C on *in situ* real-time ThT fluorescence.** Solutions of stB wt preformed fibrils were incubated at room temperature in the absence and presence of different final concentrations of antioxidants. 11.4-fold volume excess of ThT was added to each well prior to fluorescence reading. For each assay, the fluorescence of antioxidant without stB wt with ThT dye was also monitored. The excitation wavelength was set to 440 nm and the emission wavelength was set from 455 to 600 nm. Emission wavelength step size was 1 nm and excitation and emission bandwidths were set at 7.5 nm. Each sample was followed in triplicate and the mean value was presented.

Additional information about the conformational changes in the stefin B domain upon interactions with different polyphenols was obtained by circular dichroism (CD) spectroscopy as described in section 2 (Figure S2). This method is very sensitive and reliable for monitoring any conformational alteration in the protein structure upon interaction with different ligands. The far-UV region (190-250 nm) of CD spectra provides information about the secondary structure of the protein. We measured far UV CD spectra of stefin B under the conditions of amyloid fibrillation after 24 hours in presence and absence of 10 µM and 50 µM Cur, 50 µM Quer and 50 µM Res (Figure S2). Two negative bands at 208 nm and 222 nm in the far-UV region of the CD spectra of the stefin B provide information about the α-helical structure of the protein which can be explained by n → π* transition for the peptide bond of α-helix in this area. In our study addition of different polyphenol antioxidants did not induce any significant change in band intensity at 208 and 222 nm or any considerable shift of the peaks. Therefore we can conclude that polyphenols did not cause significant alteration in the secondary structure contents of the protein.

### Figure S2. The CD spectra of stB wt assemblies aged 24 hours in absence and presence of 50 µM Res, 50 µM Quer, 50 µM Cur and 50 µM Cur. Far-UV CD spectra were measured at room temperature by using a Circular Dichroism Spectrometer MOS-500 ([Bio-Logic Science Instruments](https://www.google.ba/url?sa=t&rct=j&q=&esrc=s&source=web&cd=2&cad=rja&uact=8&ved=0ahUKEwiSyJjFwfrVAhURnRQKHXfyBngQFgguMAE&url=http%3A%2F%2Fwww.bio-logic.net%2Fen%2Fproducts%2Fspectrometer%2Fmos-200-mos-200m-spectrometer%2F&usg=AFQjCNGG3HzUIZeBlaLRVfpuLQyvZLzTIg)). A 1 mm quartz cuvette was used for all CD spectra. Data were recorded from 250 to 200 nm with 1 nm sampling interval. The samples used in CD experiments were prepared similarly to those used in ThT assays except the addition of ThT dye. The final spectra were the average of three repeated experiments and the background (the CD spectrum of the sample without stB wt and antioxidants) was subtracted.

Oligomeric state of the fibrillation mixture was checked as described in section 2. All samples were subjected to crosslinking prior to applying on the SDS-PAGE gel. stB wt in absence and presence of the crosslinker were used as controls (Figure S3, Lane 1 and 2, respectively). It can be seen that the monomeric form prevails when the stB wt was applied without the BS^3^ (Figure S3, Lane 1), whereas both monomeric and dimeric forms appear when it is subjected to crosslinking (Figure S3, Lane 2). Moreover, SDS-PAGE electrophoresis has shown that antioxidants shift the equilibrium to higher forms such as tetramers and higher oligomers (Figure S3. tetramers with the molecular mass of ~44 kDa and higher oligomers are marked with arrows). According to the gel, there are more dimers (molecular mass ~22 kDa, marked with an arrow) than in the case of the control (Figure S3). Noteworthy, higher oligomers appear only when protein is exposed to very high concentrations of antioxidants, in our case 2.5 mM and 6 mM vit C (Figure S3. Lane 6, 7) and 2.5 mM and 6 mM NAC (Figure S3. Lane 9 and 10).


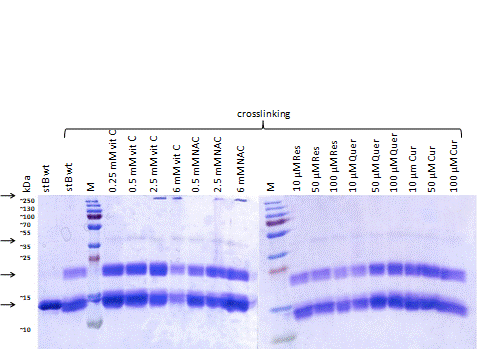


**Figure S3.** SDS–PAGE of stB wt assemblies aged 24 hours in the absence and the presence of different concentrations of antioxidants. Each sample was subjected to crosslinking with a 50-molar excess of BS^3^ except for the lane 1 where fresh stB wt was applied without crosslinker.

Molecular docking study using SwissDock has offered predictions of binding modes for each antioxidant (Figure 10, Supplementary data: Table S1). Databank was used to determine amino acid residues that participate in specific protein-ligand interactions according to predictions (Supplementary data: Table S1). Table S1 shows amino acid residues that participate in interactions and interactions which are probably involved in the binding regime. Interestingly, the program has offered many prediction versions for each antioxidant with exception of curcumin; this antioxidant has each time been positioned within the same pocket (Figure 10E, Supplementary data: Table 1). On top of that, according to docking parameters, the estimated ΔG value is the lowest for curcumin suggesting the strongest binding (Table S1). This observation is supported by results of quenching intrinsic Tyr fluorescence (Supplementary data: Figure S4E, S5B, D). Supplementary figure S4E shows a significant decrease of Tyr fluorescence intensity in a concentration-dependent manner, whereas Stern-Volmer plots and binding constants show that curcumin binds more tightly than other antioxidants studied here (Supplementary data: Figure S5B, D).

**Table S1.** Molecular docking parameters of antioxidants-protein interaction.

| Ligand | Amino acid residues | Interactions involved | Estimated ΔG (kcal/mol) |
| --- | --- | --- | --- |
| NAC | Pro^36^, Val ^37^, Val^57^, His ^58^, Val^59^, Gly^60^ | Hydrophobic and  H-bonding | -6.96 |
| Vitamin C | Asp^61^, Glu^62^, Asp^63^ | H-bonding | -7.16 |
| Resveratrol | Asp^63^, Phe^64^, Val^65^, Ala^90^ | Hydrophobic and  H-bonding | -6.42 |
| Quercetin | Phe^35^, Pro^36^, His^58^, Val^59^, Gly^60^ | Hydrophobic and  H-bonding | -6.91 |
| Curcumin | Val^59^, Gly^60^, Asp^61^, Glu^62^, Asp^63^  Lys^89^, Ala^90^, Lys^91^, His^92^, Asp^93^ | Hydrophobic and  H-bonding | -7.89 |

More information about each antioxidant is given in the Table S2. When compared to other ligands included in this study, curcumin has a specific chemical scaffold (Figure 1E); it contains two substituted aromatic groups symmetrically bound by a short carbohydrate chain. Due to such structure curcumin has distinct chemical and physical properties among other antioxidants (Table S2). On the first hand, it can act as both hydrogen bond donor and acceptor. This property does not seem to be crucial for its interactions with stefin B as other ligands have a significant potential for acting as both donors and acceptors, as well (Table S2), but is probably important. On the other hand, it has a significantly longer linker, higher rotatable bond count, and partition coefficient, that probably contribute to its specific behavior. Among other ligands, resveratrol is the most lipophilic as its partition coefficient is 3.1, followed by quercetin (XlogP3 = 1.5) but their rotatable bond counts are much lower, i.e. 2 and 1, respectively, and linkers are shorter, as well. Vitamin C and NAC have much lower partition coefficients (-1.6 and 0.4, respectively) and lower rotatable bond count (2 and 3, respectively). All these properties provide curcumin with many moieties which ensure specific interactions and lead to its specific positioning in a stefin B's pocket (Figure 7E).

**Table S2.** Computed Chemical and Physical Properties from <https://pubchem.ncbi.nlm.nih.gov/>

| Ligand | Hydrogen Bond Donor Count | Hydrogen Bond Acceptor Count | Rotatable Bond Count | XLogP3 |
| --- | --- | --- | --- | --- |
| NAC | 3 | 4 | 3 | 0.4 |
| Vitamin C | 4 | 6 | 2 | -1.6 |
| Resveratrol | 3 | 3 | 2 | 3.1 |
| Quercetin | 5 | 7 | 1 | 1.5 |
| Curcumin | 2 | 6 | 8 | 3.2 |

Secondly, it has already been stated that all ligands in our study act as quenchers of tyrosine fluorescence (Figure S4). More information about the design of the quenching experiment is given in section 2. According to Stern-Volmer plots, which were constructed based on quenching data and constants obtained from Stern-Volmer equations (Figure S5), it is evident that in this pool of compounds Cur has the tightest connections with stefin B, which create a strong network and lead to alterations of its amyloid fibrillation profile. Moreover, these plots also show that only the quenching data obtained for NAC and Cur fit to linear regression, whereas data obtained for vit C, Res and Quer do not (Figure S5A, B). Namely, a linear Stern-Volmer plot is indicative of a single class of fluorophore, which are equally accessible to the quencher. If more fluorophore populations are present and either of them is not accessible to the quencher, then the plot deviates from the linearity. This notice is frequently seen in case of charged or polar as they cannot fully penetrate into the core of the protein and therefore quench only fluorophores which are exposed on the surface of the protein. In our case, this notice is probably caused by the fact that each ligand binds differently, i.e. more or less close to Tyr residues and therefore quenching effects are not strictly concentration dependent for each of them. This interpretation is in good correlation with docking predictions (Table S1). To be more precise, Cur probably binds in the vicinity of all three Tyr residues whereas NAC binds close to Tyr53 which leads to quenching effect in a concentration-dependent manner. Mind that according to docking investigation other three ligands bind close to one of three Tyr residues as well, but they are probably oriented in such way that they quench Tyr fluorescence but without obvious concentration dependence.

**Figure S4. Steady-state fluorescence quenching measurements of stB wt in presence of different concentrations of quenchers, i.e. antioxidants.** Fluorescence spectra of stB wt sample (34 µM) were recorded with increasing concentrations of (A) NAC, (B) vit C, (C) Res, (D) Quer and (E) Cur. Excitation wavelength was 277 nm and emission wavelength was set from 290 to 360 nm. Emission wavelength step size was set to 1nm whereas excitation and emission bandwidth was 7.5 nm.

According to Figure S4 it is obvious that each antioxidant acts as quencher of intrinsic Tyr fluorescence. This finding supports docking predictions which offers specific binding modes for each quencher, i.e. antioxidant in the vicinity of one of three Tyr residues. However, Stern-Volmer plots provided us with more valuable information (Figure S5).

**Figure S5.** Stern-Volmer plots and modified Stern-Volmer plots of fluorescence quenching constant for the antioxidants-protein complexes. (A, B) Stern-Volmer plots versus different quencher, i.e. antioxidant concentration. K_sv_ constant was calculated from the equation of F_0_/F=1+K_sv_[Q] and the results are discussed in the text. (C, D) The plot of log(F_0_-F/F) as a function of logarithmic value of quencher concentration.

In other words, Stern-Volmer plots offer information regarding the number of binding sites *n* for each antioxidant. This value is presented as bolded for each compound in the Figure S5 C, D together with chart equation. According to these plots, the *n* value is the greatest for curcumin (Figure S5 D) which again points to its specific binding properties. Moreover, this value supports other findings regarding ThT fluorescence, TEM images and docking predictions where curcumin showed the greatest inhibitory effect and different binding capacity when compared to other antioxidants.
